# Severity of enterovirus A71 infection in a human SCARB2 knock-in mouse model is dependent on infectious strain and route

**DOI:** 10.1038/s41426-018-0201-3

**Published:** 2018-12-05

**Authors:** Junping Zhu, Ning Chen, Shuya Zhou, Kai Zheng, Lin Sun, Yuxiao Zhang, Lina Cao, Xiaoyan Zhang, Qiaoyan Xiang, Zhiyun Chen, Chenfei Wang, Changfa Fan, Qiushui He

**Affiliations:** 1 Department of Medical Microbiology, Capital Medical University, Beijing, 100069 China; 2 Division of Animal Model Research, Institute for Laboratory Animal Resources, National Institutes for Food and Drug Control, Beijing, 100050 China; 3 Department of Medical Microbiology and Immunology, University of Turku, Turku, 20520 Finland

## Abstract

Enterovirus A71 (EV-A71) is a major etiological agent of human hand, foot and mouth disease, and it can cause severe neurological complications. Although several genotypes of EV-A71 strains are prevalent in different regions of the world, the genotype C4 has circulated in mainland China for more than 20 years. The pathogenicity of different EV-A71 clinical isolates varies and needs to be explored. In this study, hSCARB2 knock-in mice (*N* = 181) with a wide range of ages were tested for their susceptibility to two EV-A71 strains with the subgenotypes C4 and C2, and two infection routes (intracranial and venous) were compared. The clinical manifestations and pathology and their relationship to the measured viral loads in different tissues were monitored. We observed that 3 weeks is a crucial age, as mice younger than 3-week-old that were infected became extremely ill. However, mice older than 3 weeks displayed diverse clinical symptoms. Significant differences were observed in the pathogenicity of the two strains with respect to clinical signs, disease incidence, survival rate, and body weight change. We concluded that hSCARB2 knock-in mice are a sensitive model for investigating the clinical outcomes resulting from infection by different EV-A71 strains. The intracranial infection model appears to be suitable for evaluating EV-A71 neurovirulence, whereas the venous infection model is appropriate for studying the pathogenicity of EV-A71.

## Introduction

Hand, foot and mouth disease (HFMD) is a common childhood disease, that is characterized by rapidly ulcerating vesicles in the mouth and vesicular lesions on the hands, feet, and buttocks. In China, HFMD has exhibited the highest morbidity and mortality among the group C notifiable infectious diseases in the last decade (http://www.nhfpc.gov.cn/jkj/s3578/new_list.shtml). Enterovirus A71 (EV-A71) is a member of family Picornavieidae that is one of major etiological agent of HFMD and causes a variety of clinical manifestations including severe neurological complications including aseptic meningitis, brainstem encephalitis, neurogenic pulmonary edema, acute flaccid paralysis, and death^1–3^. EV-A71 was first isolated from a child with encephalitis in California, USA, in 1969^4^. In the Asian-Pacific region, EV-A71-related outbreaks have been reported frequently and are associated with increased neurovirulence and fatalities^5,6^. The dominant EV-A71 strains circulating in different countries and regions vary genetically. In mainland China, more than 600,000 HFMD cases and 126 deaths were reported in Fuyang City of Anhui province from March 2008 to June 2009. Subsequently this outbreak spread quickly to other regions and caused nationwide HFMD epidemics. An emerging recombinant EV-A71 C4 genotype was reported to be responsible for this outbreak^7^. We have reported on the prevalence of EV-A71 during the HFMD epidemic in Beijing from 2007 to 2009, including the isolation of the CMU4232 strain, one of the circulating strains identified in 2008^8^. Xing et al. reported that more than 90% of deaths were associated with EV-A71 in China from 2008 to 2012^9^. However, the pathogenesis of EV-A71 infections is not fully understood, especially the cause of the severe neurological manifestation.

Numerous animal models have been developed to study the pathogenesis of EV-A71 infection, and appropriate animal models are needed to better understand EV-A71-associated neuropathogenesis and to facilitate the development of effective vaccines and drugs. EV-A71-infected cynomolgus and rhesus monkeys develop similar clinical symptoms as those observed in humans, including neurological complications^10–12^. However, the application of EV-A71 nonhuman primate models is limited because of ethical and economic reasons. Mice models with the strategies of virus adaption and immunodeficient hosts are also developed, although they do not closely mimic human disease^13–15^. Moreover, mice that are more than 2-week-old are generally not susceptible to EV-A71.

Human scavenger receptor class B member 2 (hSCARB2) is widely expressed in many human tissues and cell types, including neurons in the central nervous system (CNS)^16^. Studies have showed that hSCARB2 serves as a functional receptor for EV-A71 in vitro and in vivo^17–21^. Although the mouse SCARB2 shares 85.8% homology to the human protein, it does not function as a receptor for EV-A71^18^. To date, four reported transgenic (Tg) mice carrying hSCARB2^19,20,22–24^ have been established. Tg mice developed by Lin et al., appeared to be susceptible to EV-A71 up to 2 weeks old and exhibited pathological features similar to a suckling mouse model^20^. In their model, B genotypes of clinically isolated EV-A71 led to HFMD-like diseases while C genotypes led to neuropathogenesis, such as limb paralysis (LP). Another hybrid (hSCARB2+/+/stat-1−/−) mouse strain established from crossbreeding SCARB2 transgenic and stat-1 knockout (KO) mice was also susceptible to EV-A71 infection up to 2 weeks of age^23^. In the murine model developed by Fujii et al., young hSCARB2 Tg mice (3-week-old) were susceptible to infection by the EV-A71 Isehara strain (C genotype) and displayed features of neuropathology^19^. A recent hSCARB2 knock-in (KI) mouse model established by Zhou et al. showed that the KI mice are not only susceptible to luciferase (Luc) recombinant EV-A71 pseudovirus infection (up to 12 weeks of age) but also exhibited partial HFMD clinical symptoms present in humans, combining with bioluminescent imaging (BLI) technique^24^. In this study, we characterized of this hSCARB2 KI mice challenged by two clinical strains, including EV-A71-CMU4232, an endemic strain isolated in mainland China in 2008 and a clone-derived virus (CDV)-Isehara, whose original strain (Isehara/Japan/99) was among the circulating strains identified in Japan in 1999^25^ and was used in another hSCARB2 Tg mouse model to study EV-A71 neuropathogenesis^19^. We assessed the utility of these mice as appropriate small animal models for evaluating the pathogenicity and virulence of different EV-A71 strains.

## Results

### Characterization of CMU4232 and CDV-Isehara

**Fig. 1:**
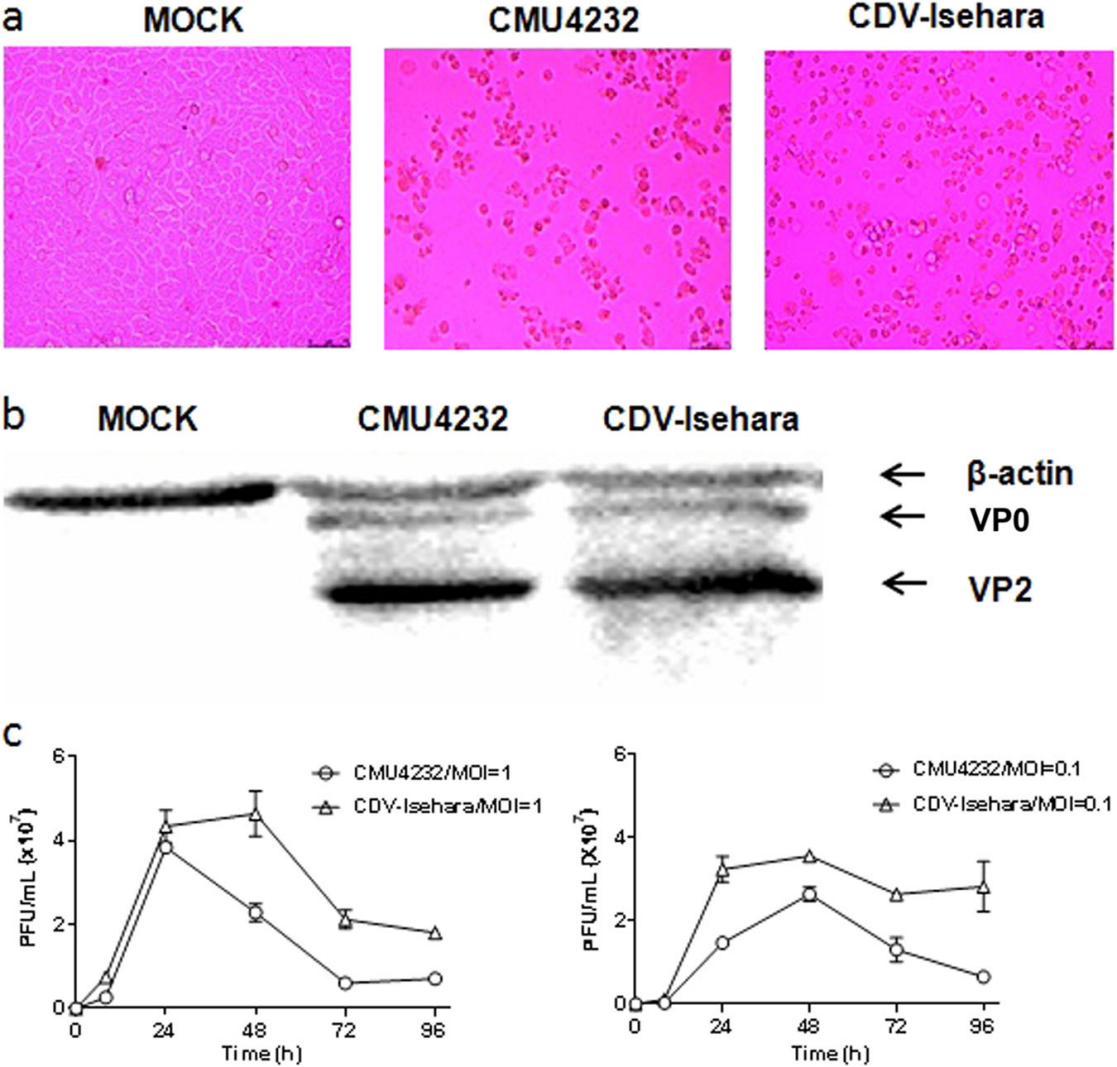
Characterization of CMU4232 and CDV-Isehara. **a** Cytopathic effects at 1dpi displayed on RD cells infected with CMU4232 or CDV-Isehara. **b** Detection of viral structural proteins VP0 and VP2 of CMU4232 and CDV-Isehara by western blotting with antibody specific for EV-A71. β-actin was used as a internal control. **c** Growth curves of CMU4232 and CDV-Isehara in RD cells. RD cells were inoculated with CMU4232 or CDV-Isehara as indicated at either MOI of 1 or 0.1. Samples were collected at the times indicated and titrated by PFU assay. All assays were performed in triplicate. At each time point, titer values are means of three samples; error bars represent SEM. dpi days post infection, PFU plaque forming unit, SEM standard error of mean

At 24-h post infection, the rhabdomyosarcoma (RD) cells infected with CMU4232 and CDV-Isehara displayed typical CPE, such as cell rounding, aggregation, detachment, and floatation (Fig. 1a). The production of specific VP0 and VP2 proteins (Fig. 1b) was detected in the lysates of the cells infected by CMU4232 or CDV-Isehara.

We next determined the growth kinetics of the two viruses in RD cells. The CMU4232 strain showed a similar proliferation rate to that of CDV-Isehara during the 24 h post-inoculation period when an multiplicity of infection (MOI) of 1 was used to inoculate the RD cells (Fig. 1c). The CMU4232 strain reached a peak viral titer (3.8 × 10^7^  plaque forming unit (pfu)) after 24 h and then decreased, whereas CDV-Isehara reached a peak viral titer (4.3 × 10^7^ pfu) at 48 h post-inoculation. We also determined the growth curve for both viruses at an MOI of 0.1. At a low MOI, CMU4232 and CDV-Isehara reached a peak viral titer at 48 h postinoculation. The maximum viral titer observed for CMU4232 and CDV-Isehara was 2.6 × 10^7^ pfu and 3.55 × 10^7^ pfu, respectively (Fig. 1c). These data indicated that the CDV-Isehara strain replicated relatively faster than that of the stain CMU4232 in RD cells.

### Sequences and phylogenetic analyses of the strains CMU4232 and CDV-Isehara

**Fig. 2:**
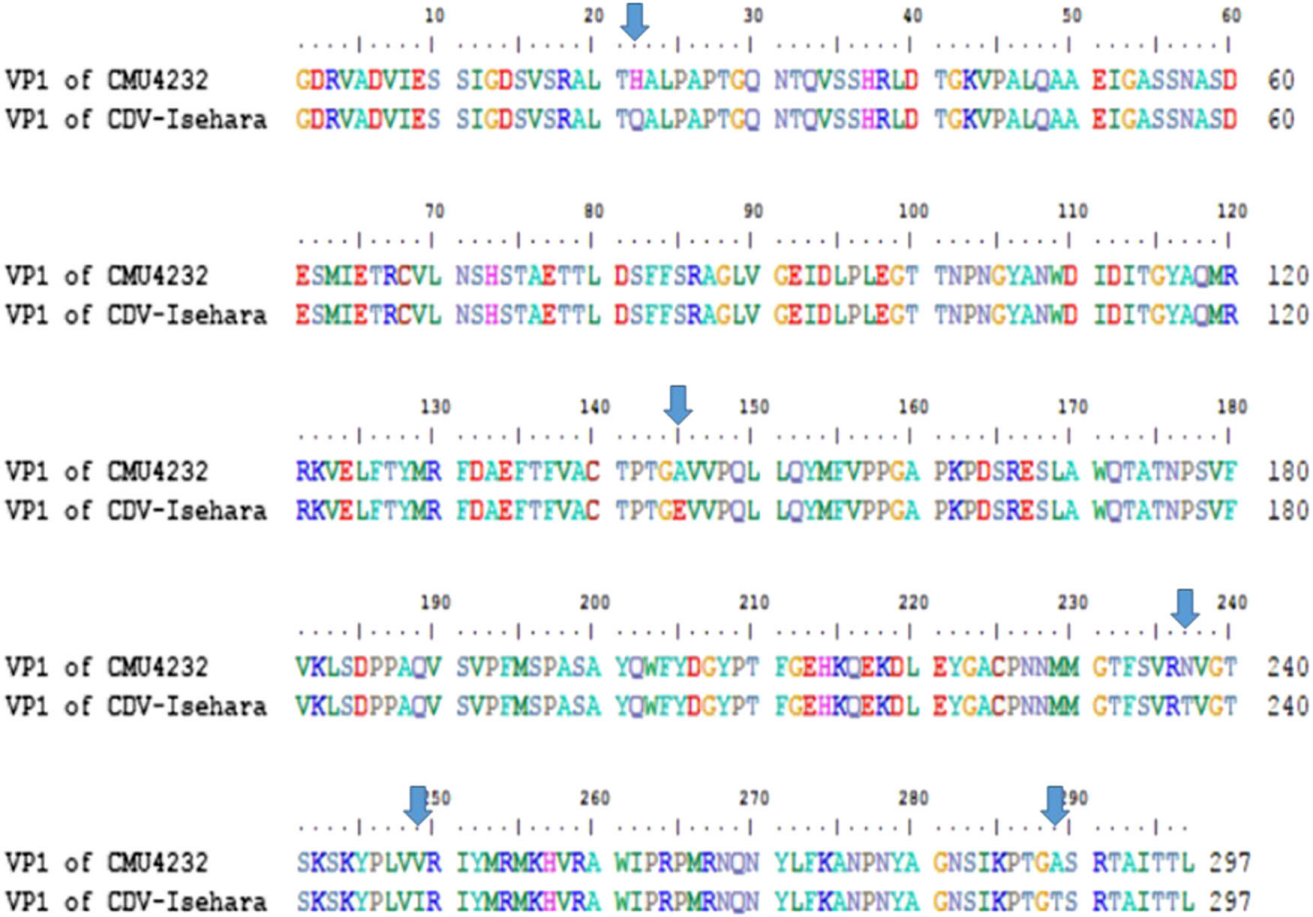
Variation of the VP1 amino acid sequences between CMU4232 and CDV-Isehara strains. In total, there were 297 amino acids in the VP1 protein of EV-A71, and five residue variations (22 H/Q, 145 A/E, 237 N/T, 249 V/I, and 289 A/T) were found in the VP1 amino acid sequence between CMU4232 and CDV-Isehara strains

The complete genomes of CMU4232 and CDV-Isehara exhibited a nucleotide similarity of 83%. The nucleotide and amino acid similarities of the four structural genes (VP1 to VP4) between CMU4232 and CDV-Isehara were observed to be 89–90.8% and 98.3–100%, respectively (S1 Table). Of the 297 amino acids of VP1 protein, five substitutions were identified, including H22Q, A145E, N237T, V249I, and A289T (Fig. 2), whereas only one substitution (I31M) was observed in the 254 amino acids of the VP2 protein. No substitutions were observed in the VP3 and VP4 proteins between the two strains (S1 Table).

**Fig. 3:**
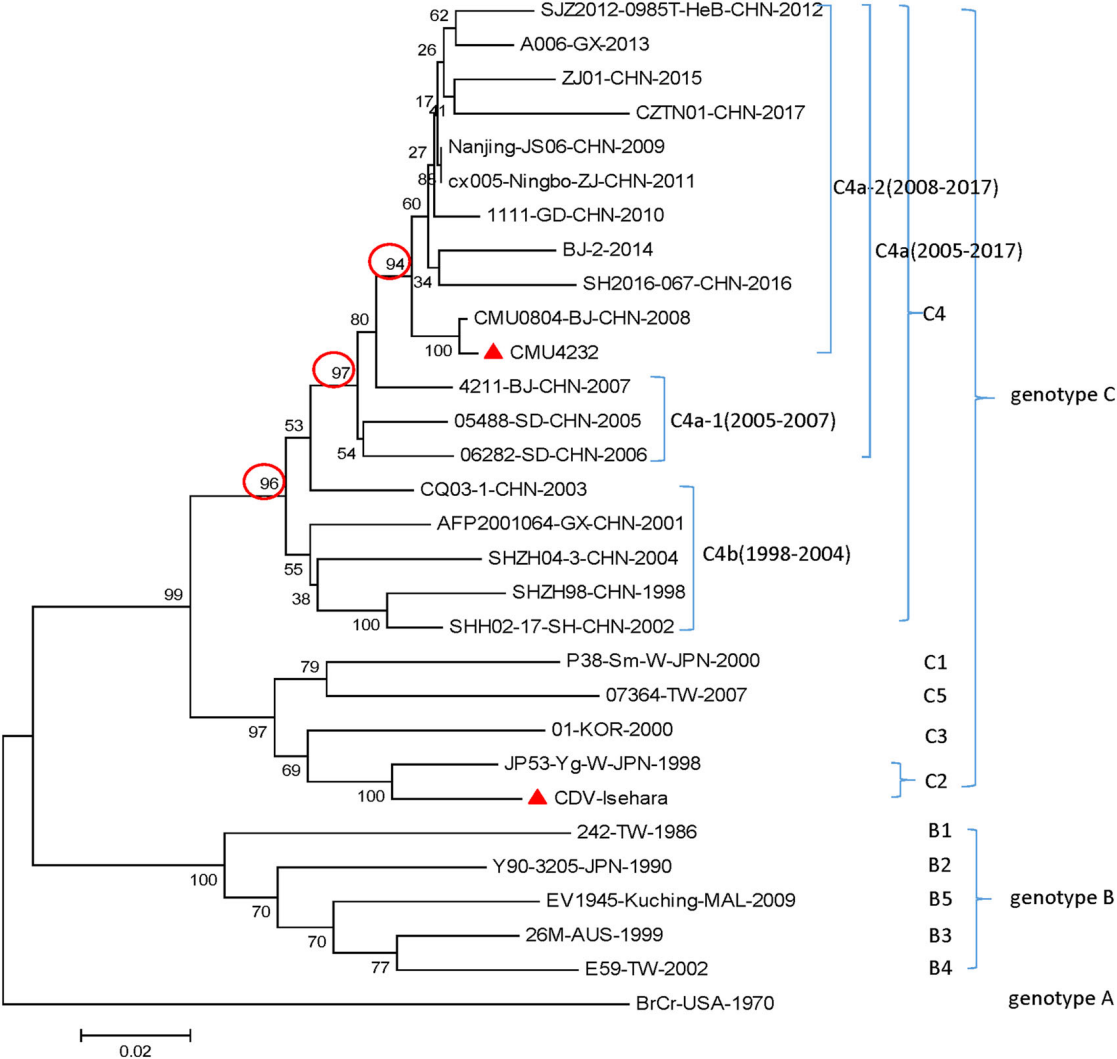
Phylogenetic relationship among 30 worldwide EV-A71 strains based on the complete VP1 gene. CMU4232, CDV-Isehara, 18 representative CHN strains, and 10 international representative strains of different subgenotypes are included in this dendrogram. The 18 representative CHN strains were selected from different isolation places in mainland China from 1998 to 2017 according to available data on internet. Details of all the EV-A71 strains included in the dendrogram are provided in Table S1. CHN Chinese

In the phylogenetic analysis, the sequences of 30 prevalent EV-A71 strains reported from different regions of the world were compared, including the strains CMU4232 and CDV-Isehara used in this study. Consistent with the results of previous studies, all the representative strains isolated in China during 1998–2017 were clustered into the C4 genotype. The C4 cluster showed a stepwise evolutionary trajectory over time. There are two branches included in the genotype C4, the early C4b (1998–2004) and the later C4a (2005–2017). The C4a branch could be further divided into two separate groups, C4a-1 and C4a-2, which include strains isolated from 2005–2007 and 2008–2017, respectively. The results clearly show that CMU4232 belongs to the C4a-2 of genotype C4 and CDV-Isehara belongs to genotype C2 (Fig. 3).

### hSCARB2 KI mice are susceptible to CDV-Isehara intracerebrally (I.C.) infection

**Fig. 4:**
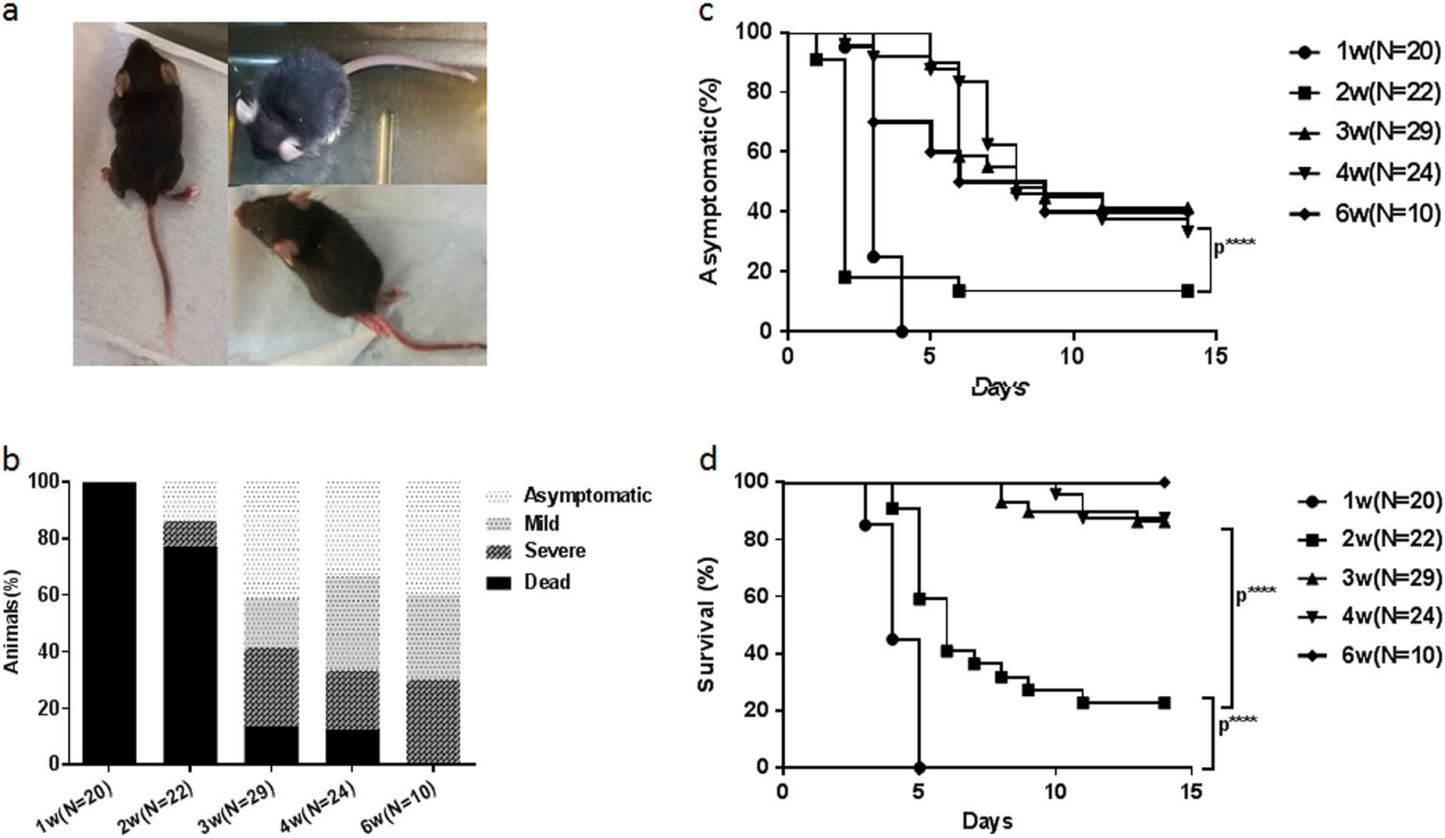
Susceptibility of hSCARB2 KI mice to CDV-Isehara infection. The 1, 2, 3, 4, and 6-week-old hSCARB2 Tg mice were infected intracerebral (I.C.) with CDV-Isehara strain at a dose of 4.8 × 10^6^ pfu and monitored daily after infection. **a** The CNS-like hindlimb paralysis, ruffled fur, and shrinking. **b** The clinical symptoms (asymptomatic, mild, severe, and dead), followed by the criteria described in the materials and methods section (**c**) asymptomatic (%) and (**d**) survival (%). Significant difference of asymptomatic (%) and survival (%) with different weeks old Tg mice infected with CDV-Isehara was shown as ****p,0.0001. pfu plaque forming unit, CNS central nervous system, I.C. intracerebral

When hSCARB2 KI mice of different ages were challenged, 76/105 showed ataxia, paralysis, or death at days 2–14 after infection (Figs. 4a, b and Supporting videos). A significant difference in the disease incidence and death rate was observed between KI mice younger and older than 3 weeks (*P* < 0.0001) (Figs. 4c, d). In addition, the death rate of 1-week-old-infected KI mice was 100% (20/20) from 3 to 5 days post inoculation (dpi), while that of 2-week-old-infected Tg mice was 77.3% (17/22) from 4 to 11 dpi (*P* < 0.0001) (Fig. 4d). Paralysis occurred in both the forelimb and hindlimb post infection, particularly in the hindlimb. Some of the KI mice died within 1–3 days after LP symptoms appeared, while some were still alive when the symptoms gradually decreased. The susceptibility of the hSCARB2 KI mice to CDV-Isehara infection decreased as the age of mice increased, although no significant difference was observed among groups of KI mice older than 3 weeks (*P* > 0.05) (Figs. 4b–d). The development of disease symptoms in 4-week-old-infected KI mice was very similar to that of 3-week-old (Fig. 4b). In 4-week-old-infected KI mice, 33.3% (8/24) and 20.8% (5/24) experienced mild and severe symptoms, respectively, while 12.5% (3/24) died, whereas 17.2% (5/29) and 27.6% (8/29) of 3-week-old-infected KI mice exhibited mild and severe symptoms, respectively, while 13.8% (4/29) died.

### Difference in clinical features of hSCARB2 KI mice challenged by CMU4232 and CDV-Isehara

**Fig. 5:**
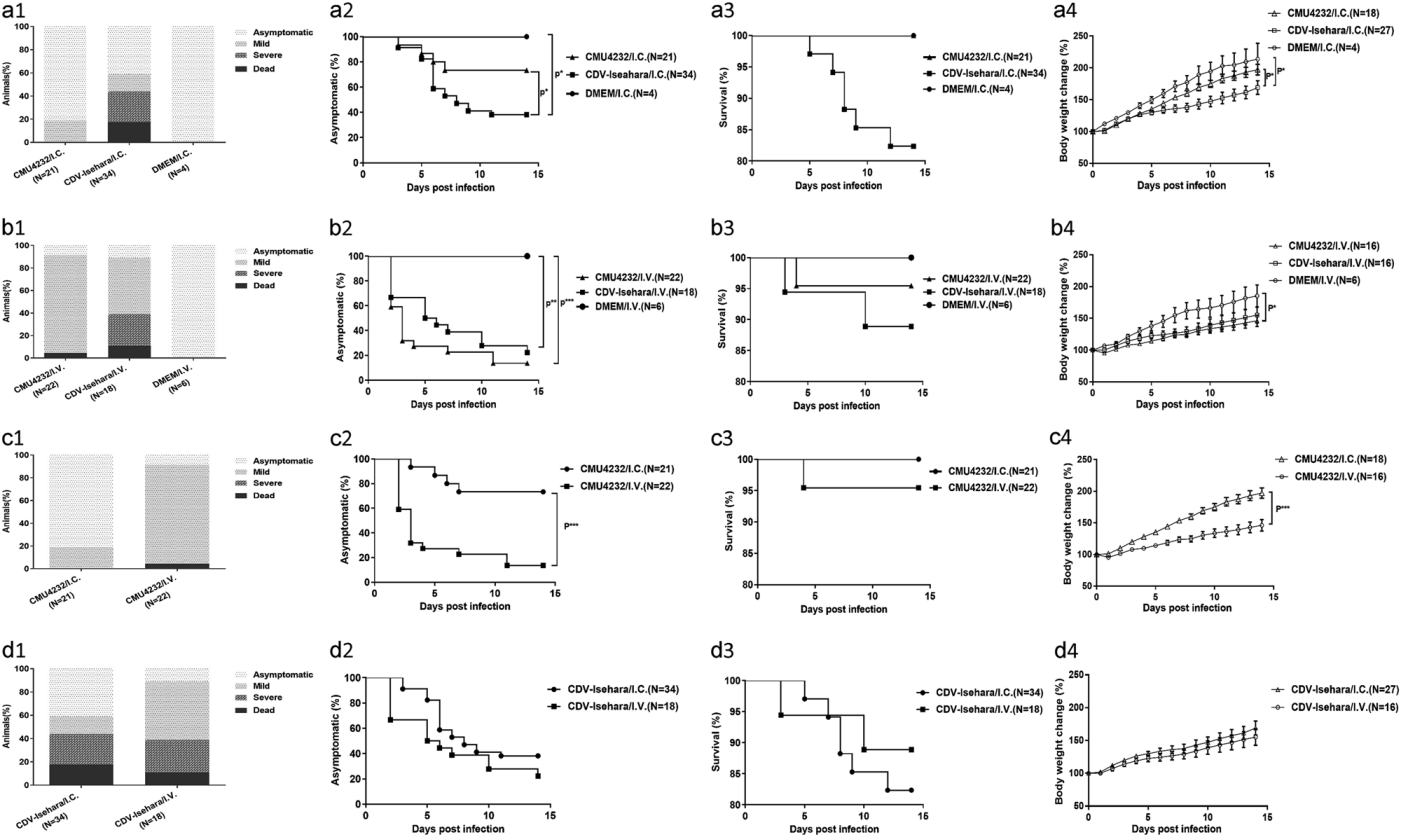
The clinical symptoms, asymptomatic (%), survival (%), and body weight change (%) of different EV-A71 Tg mouse models. Three-week-old hSCARB2 KI mice were inoculated I.C. with DMEM/ EV-A71 (**a**) or inoculated intravenous (I.V.) with DMEM/EV-A71 (**b**) respectively. Three-week-old hSCARB2 KI mice were inoculated with CMU4232 (**c**) /CDV-Isehara (**d**) strain via I.C. or I.V. route respectively. (1) The clinical symptoms, (2) asymptomatic (%), (3) survival (%), and (4) body weight change (%) of the Tg mice were observed and recorded daily for 2 weeks after viral infection. ***p,0.000. I.C. intracerebral, I.V. intravenous

Differences in the clinical features of hSCARB2 KI mice caused by the CMU4232 and CDV-Isehara strains when using the same route of infection (I.C. or I.V.) were assayed for using 3-week-old hSCARB2 KI mice. Some of the KI mice infected with both EV-A71 strains led to diverse clinical signs and various degrees of manifestations, especially in the development of neurological symptoms. The peak period of disease onset was 3–7 dpi, and death was typically delayed by 1–2 days after this period. No clinical signs were observed in the mock-infected KI mice (Fig 5a1, a2, b1, and b2).

The death rate of the CDV-Isehara/I.C. group was 17.6% (6/34), and 41.2% (14/34) of mice displayed mild or severe clinical signs, including ruffled fur, hunchbacked appearance, easily frightened, trembling, shrinking, limb weakness, mental bluntness, dispirited, slow in action, emaciation, and LP, whereas only mild symptoms (19%) occurred in the CMU4232/I.C. group (*N* = 21), with trembling, shrinking, and limb weakness observed, and these mild symptoms completely disappeared soon thereafter (Fig 5a1).

The clinical signs of the CDV-Isehara/I.C. group were observed in 20 of 34 mice from 3 to 11 dpi, while clinical signs were observed in 4 of 21 mice from 3 to 7 dpi for the CMU4232/I.C. group (Fig 5a2). A significant difference in disease incidence was observed between these two groups (*P* < 0.05) (Fig 5a2). However, no significant differences were found in death rate between them (*P* > 0.05) (Fig 5a3). Among the groups infected via the I.C. route, a significant difference in body weight change was also observed (*P* = 0.023) (Fig 5a4). Furthermore, significant differences were observed between the CDV-Isehara/I.C. and CMU4232/I.C. or DMEM/I.C groups (*P* = 0.042 and *P* = 0.047, respectively) (Fig 5a4). The body weights of mice in the CDV-Isehara/I.C. group increased much slower than those of in the CMU4232/I.C. and DMEM/I.C. groups, demonstrating that CDV-Isehara infection affected the growth of infected KI mice more than CMU4232 when administered via the same I.C. route. Taken together, the disease severity induced by the strain CMU4232 in 3-week-old hSCARB2 KI mice was less than that caused by the strain CDV-Isehara. These results suggested that these KI mice were susceptible to EV-A71 and that there was a difference in sensitivity of the hSCARB2 KI mice to the two strains.

When the onset of typical symptoms was compared, hSCARB2 KI mice infected with the strain CMU4232 via the I.V. route exhibited typical symptoms from 2 to 11 dpi, including ruffled fur, hunchbacked appearance, easily frightened, trembling, shrinking, limb weakness, mental bluntness, dispirited, slow in action, emaciation, and death, but no severe symptoms or paralysis occurred when the mice were followed up to 14 dpi. However, the symptoms and consequences of CDV-Isehara/I.V. infection were more severe from 2 to 14 dpi, with paralysis in the limbs and death observed in this group (Fig 5b1, b2).

In the hSCARB2 KI mice I.V. infected with CMU4232, 86.4% (19/22) developed mild symptoms and 4.5% (1/22) died whereas 50% (9/18) and 27.8% (5/18) of mice in CDV-Isehara/I.V. group exhibited mild and severe symptoms, respectively, while 11.1% (2/18) died (Fig 5b1). A significant difference in the disease incidence was observed between the CMU4232/I.V. or CDV-Isehara/I.V. groups and the DMEM/I.V. group (*P* < 0.0005 and *P* < 0.005, respectively) (Fig 5b2). However, no significant differences in disease incidence and death rate were observed between CMU4232/I.V. and CDV-Isehara/I.V. groups. In the CDV-Isehara/I.V. group, two of 18 infected Tg mice died from 3 to 10 dpi, while one death was observed in the CMU4232/I.C. group at 4 dpi (Fig 5b3). Furthermore, no difference in body weight change was observed between these two groups (*P* = 0.468), although a significant difference was observed between the CDV-Isehara/I.V. and DMEM/I.V. groups (*P* = 0.012) (Fig 5b4).

We next compared the disease development of hSCARB2 KI mice infected with the same strain via different routes (I.C. or I.V.). For KI mice infected with CMU4232, distinct symptoms were observed and were strongly dependent on the infection route. The severity of the disease caused by the I.V. route was more severe than that of I.C. route based on the observed clinical symptoms, disease incidence (*P* < 0.005) and the body weight change (*P* < 0.005) (Fig 5c1–c4). However, the severity of the disease induced by CDV-Isehara was similar for both infection routes, irrespective to the observed clinical manifestations, disease incidence, death rate, and the body weight change (*P* > 0.05) (Fig 5d1–d4).

### Viral proliferation and pathological features in hSCARB2 KI mice

**Fig. 6:**
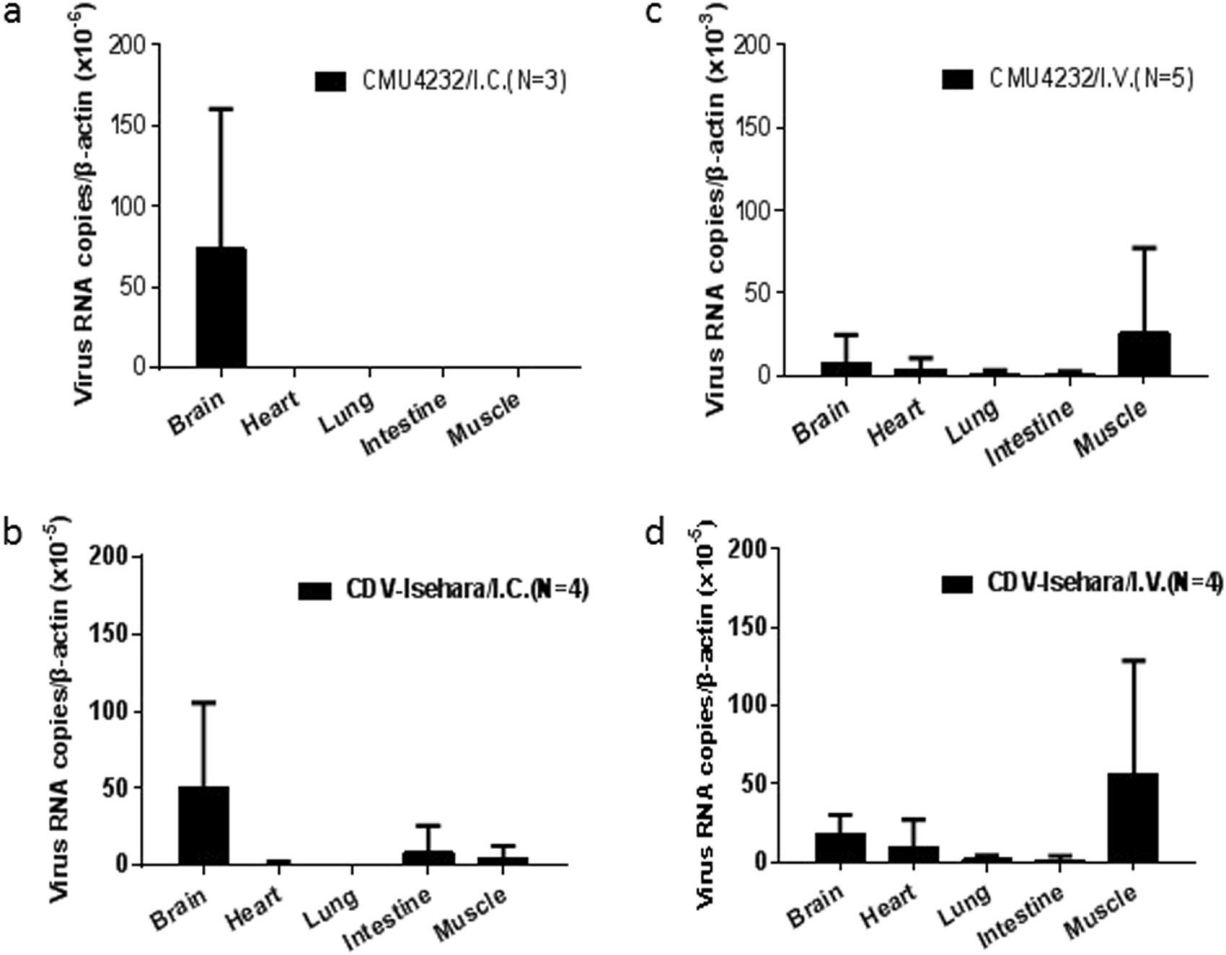
Virus replication in some tissues of hSCARB2 KI mice by EV-A71 infection. Three-week-old hSCARB2 KI mice were inoculated with CMU4232/ CDV-Isehara via I.C. or I.V. route. EV-A71 genome copy numbers in the brain, heart, lung, intestine, and muscle of EV-A71-infected Tg mice were determined at several time points by quantitative RT-PCR. Mouse β-actin gene expression in each tissue was used as the internal control. Results represent the mean of three to five samples; error bars represent STDEV. **a** CMU4232/I.C. (*N* = 3); **b** CDV-Isehara/I.C. (*N* = 4); **c** CMU4232 /I.V. (*N* = 5); **d** CDV-Isehara/I.V. (*N* = 4)

To assess viral proliferation in vivo, the viral RNA in tissues of infected 3-week-old KI mice was quantified by real-time PCR (Fig. 6). Specific viral RNAs were detected in the brain of CMU4232/ I.C. KI mice (Fig. 6a), while in the CDV-Isehara/I.C. group, viral RNAs were detected in the brain, intestine, muscle, and heart tissues of mice, but no viral RNAs were identified in the lungs (Fig. 6b). The distribution of both EV-A71 strains in tissues of KI mice infected via the I.V. route was highly consistent. However, higher viral loads were detected in CMU4232-infected KI mice than was observed in CDV-Isehara-infected mice. Furthermore, the viral load in the muscle was higher than that observed in the brain, heart, lung, and intestine, demonstrating the susceptibility of different tissues of hSCARB2 KI mice to EV-A71 when infected via the I.V. route (Figs. 6c, d). No viruses were detected in any tissue of the control group, which was inoculated with RD cell lysate supernatant.

Next, we observed the pathological changes in the two major replication sites, the brain and skeletal muscle (Fig. 6). Different degrees of pathological changes were evident in the KI mice with clinical signs. These changes primarily included cellular damage (such as necrosis and neuronophagia) and inflammatory changes (such as gliosis and perivascular cuffing) (Fig 7.1–7.3) in the brain. In addition, degeneration and necrosis in muscle bundles and severe necrotizing myositis with a mass of inflammatory cell infiltration was observed (Fig 7.5–7.7), especially in KI mice infected with CDV-Isehara via the I.V. route. Viral antigens were also detected in the above affected tissues. Immunohistochemistry (IHC) staining revealed the presence of viral antigen in the tissues with pathological changes, indicating that the pathological findings were caused by EV-A71 infection. All mock mice tested negative for viral antigens, regardless of the infection routes (Fig. 8).

**Fig. 7:**
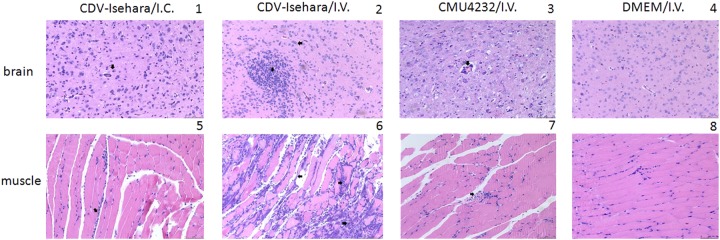
Pathological analysis of brain and the skeletal muscle of EV-A71-infected mouse models. Sporadic neuronal necrosis and neuronophagia (arrowhead) in the brain (1) and Inflammatory cell infiltration (arrowhead) in the skeletal muscle (5) of mouse model with CDV-Isehara/I.C.; glial nodule (arrow) and neuronophagia (arrowhead) in the brain (2) and severe necrotizing myositis with a mass of inflammatory cell infiltration (arrow) in the skeletal muscle (6) of mouse model with CDV-Isehara/I.V.; perivascular cuffing (arrowhead), partial neuronal necrosis and neuronophagia in the brain (3) and degeneration and necrosis in muscle bundles with inflammatory cell infiltration (arrowhead) (7) in mouse model with CMU4232/I.V.; the brain (4) and the skeletal muscle (8) of MOCK mouse with DMEM/I.V. (magnification: 200 × ). Representative samples are shown for each group

**Fig. 8:**
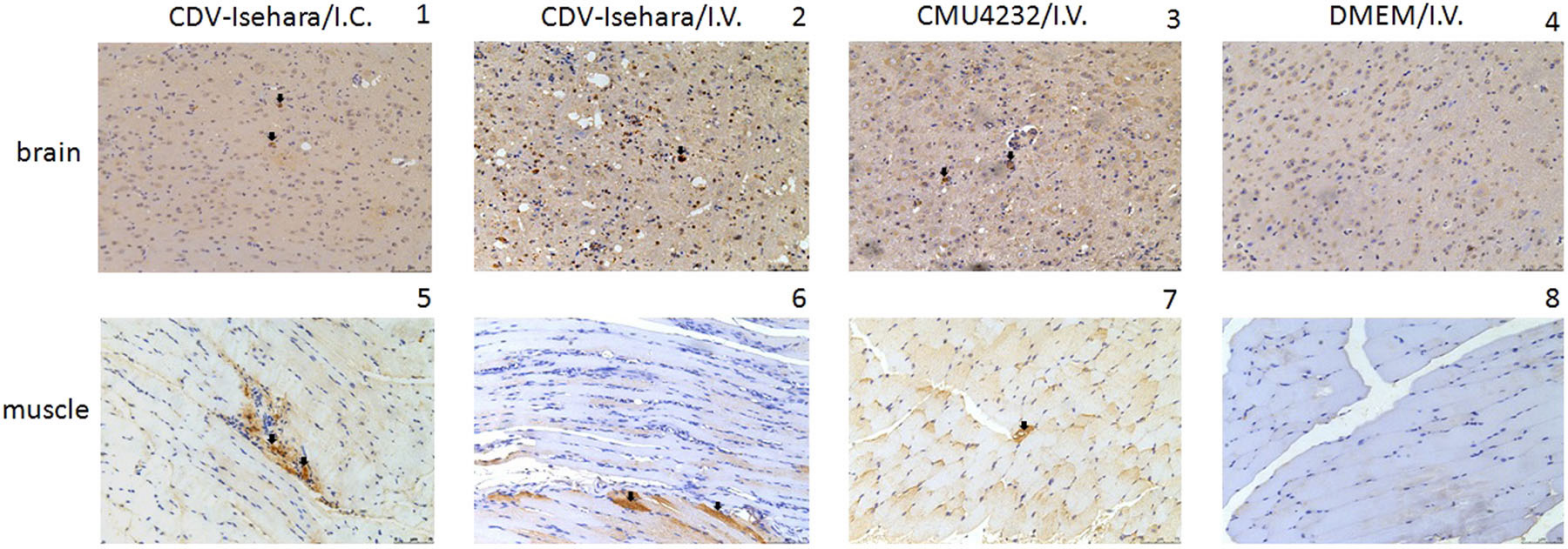
VP2 expression in the brain and the skeletal muscle of EV-A71-infected mouse models. The brain (1) and the skeletal muscle (5) of mouse model with Isehara/I.C.; the brain (2) and the skeletal muscle (6) of mouse model with CDV-Isehara/I.V.; the brain (3) and the skeletal muscle (7) of mouse model with CMU0804/I.V.; the brain (4) and the skeletal muscle (8) of MOCK mouse with DMEM/I.V. Arrowheads indicate EV-A71-positive cells (IHC staining against EV-A71 VP2, magnification: 200 × ). Representative samples are shown for each group

## Discussion

For robust results, an adequate number of hSCARB2 KI mice (*N* = 181) were used to establish a mouse model that is sensitive to EV-A71 infection in this study. In this mouse model, we observed that 3 weeks is a crucial age, as infected mice younger than 3-week-old became extremely ill. The susceptibility of the KI mice to EV-A71 infection could be observed until 8-week in mice with viral challenged by EV-A71 at 6-week-old age. Fujii et al. reported that their Tg mice older than 6 weeks were also susceptible to EV-A71 infection, and the development of disease was similar to that of 3-week-old mice^19^. The model based on 3-week-old weaning hSCARB2 KI mice in the present study displayed diverse clinical symptoms and was characterized as pathological tropism in the CNS and peripheral sites.

Based on the reliable EV-A71 disease model, we evaluated the pathogenicity of an endemic strain (CMU4232) in mainland China, and compared it with an internationally published strain (Isehara)^19^. We observed that disease manifestations induced by CDV-Isehara were more severe than those induced by CMU4232. Both the biological characteristics of specific viral strains and infection routes were observed to be important for the pathogenicity of EV-A71 in this hSCARB2 KI mouse model.

Previous studies reported that different EV-A71 strains could display varying pathogenic effects^26–28^. When infected via the I.C. route, the disease induced by CDV-Isehara was more severe than that induced by CMU4232 at the same dose (Fig 5a1) in our KI mice model. A significant difference in disease incidence between these two groups was observed (*P* < 0.05) (Fig 5a2). For mice in the CMU4232/I.C. group, only mild clinical symptoms (4/21) developed from 3 to 7 dpi, and these symptoms subsequently completely disappeared. In contrast, mice in the CDV-Isehara/I.C. group exhibited a longer onset (from 3 to 11 dpi) of various mild manifestations, paralysis, and death (6/34). In addition to the difference in disease incidence caused by the two strains, we also observed a difference in the presence of the viruses infected mouse tissues. Using qRT-PCR, viral RNAs were detected in the intestine, muscle, heart, and brain tissues of mice in the CDV-Isehara/I.C. group. We speculated that CDV-Isehara replicated well in the brain after I.C. inoculation and then entered into multiple organs via viremia, leading to viral infections that caused clinical manifestations (Fig. 6b). In contrast, viral replication of the strain CMU4232 was only detected in the brains of mice after infected via the I.C. route, and viral titers in other non-CNS organs were below detectable levels via qRT-PCR. This difference suggested that the strain CMU4232 may be less sensitive to or replicated less efficiently in the brain of this mouse model, resulting in the eventual elimination of the virus. Thus, low viremia failed to induce an effective infection in other non-CNS tissues. With I.C. administration, the brain is the most direct injury site. Both CMU4232 and CDV-Isehara strains can replicate at this site, indicating that both strains possess the characteristics of neurotropism. However, the neurovirulence of the strain CMU4232 appeared to be weaker than that of CDV-Isehara in this model. Considering that the CDV-Isehara strain originated from an infectious clone, it is unclear whether there could be some change in virulence compared with the original strain. However, the neurovirulence of the original Isehara/Japan/99 isolate in another hSCARB2 Tg mouse model was significantly superior to those of other EV-A71 strains from encephalitis and death cases^19^.

Based on a phylogenetic analysis, the two strains used in this study were determined to have the genotypes C4 and C2, respectively (Fig. 3). The different genetic backgrounds of the two strains may explain the difference in virulence observed between the two EV-A71 strains in our mouse model. We observed that the nucleotide similarity of the CMU4232 and CDV-Isehara complete genomes was only 83%.

VP1 of EV-A71 is known to be an important immunodominant protein that mediates the binding of this virus to host receptor and determines its tissue tropism. Elizabeth et al. reported that a single mutation (K244E) in the VP1 is responsible for increased virulence and neurotropism in adult interferon-deficient mice^29^. When the deduced amino acid sequences were compared in this study, difference in five residues (H22Q, A145E, N237T, V249I, and A289T) were observed between the two strains CMU4232 and CDV-Isehara (Fig. 3). The substitution of VP1-145G or -145Q for -145E has been shown to be responsible for murine adaptation and/or virulence, either alone or in combination with changes in other amino acids in different mouse models^30–33^. In our hSCARB2 KI mice, the strain CDV-Isehara harbors VP1-145E and induce more severe neurological manifestations and myositis than those induced by the strain CMU4232 which harbors VP1-145A (Figs. 7 and 8). These results suggest that EV-A71 with VP1-145E can confer better in vivo fitness of the virus, and viruse carrying this mutation are more virulent than strains with other amino acids at this position (VP1-145A/G/Q) in mouse models. Furthermore, Kataoka et al. identified VP1-145 as a critical molecular determinant for the binding of EV-A71 to another specific cellular receptor, human P-selectin glycoprotein ligand-1 (PSGL-1)^34^. The strong in vivo selection of VP1-145E variants and CNS spread have been suggested to occur in a PSGL-1-independent manner. The VP1-145E variant was primarily responsible for the development of viremia and neuropathogenesis in a cynomolgus monkey model^35^.

Previous studies showed that the amino acid VP1-145 is a variable residue among the clinical EV-A71 isolates, and it serves as a major site of positive selection in the evolution of EV-A71^3,36,37^. Molecular epidemiological studies have shown that strains with VP1-145G/Q/R are more frequently observed in severe neurological cases in humans than VP1-145E isolates^8,38–40^. Our finding also suggested that VP1-145E may be a crucial residue that is responsible for increased virulence in mice through unclear mechanisms, which may involve the other receptors and proteins required for viral entry and host immune responses to EV-A71. Two latest studies conducted in Japan reported that the amino acid VP1-145E contributed to virulence determination by controlling attachment receptor and antibody sensitivity^41,42^. The virus Isehara with VP1-145E replicated efficiently in the CNS and acted as a virulent phenotype in their hSCARB2 Tg mice. However, VP1-145G viruses were adsorbed by attachment receptors such as heparan sulfate during circulation in vivo, leading to abortive infection and less virulent phenotype in the same Tg mice model. The maintenance of viremia titer at a high level is necessary for virus entrance into CNS via the intravenous route^41^. When cynomolgus monkey model was tested, VP1-145E, but not VP1-145G viruses induced neurological symptoms. VP1-145E viruses were frequently detected in the tissues of infected monkeys, but VP1-145G viruses were detected less frequently and disappeared quickly. Clearly, VP1-145E viruses were more resistant to neutralizing antibodies than VP1-145G viruses^42^. Our results are in line with the two studies. However, the exact mechanism of the CMU4232 with VP1-145A in the infected hSCARB2 KI mice remains to be elucidated.

The difference between the pathogenicity of EV-A71 in animals and humans caused by the VP1-145 variation may partly explain why the Isehara/Japan/99 strain from a HFMD patient was observed to be more pathogenic than other clinical strains from encephalitis and death cases in another hSCARB2 Tg mouse model^19^.

In addition to the VP1-145, two other variable sites observed in this study (VP1-22 and VP1-237) have been reported to be under positive selection pressure^3,36,39^. We also observed a difference at VP2-I31M between these two strains. Huang et al. reported that amino acid changes in both A145E of VP1 and K149M of VP2 contributed to viral infectivity in vitro and mouse lethality in vivo^32^. However, we did not observe an amino acid change at 149 K of VP2.

It is known that both alanine (A) and glycine (G) are nonpolar neutral amino acids. However, it is not known whether CMU4232 VP1-145A could be adsorbed by heparan sulfate in tissues with abundant blood supply under the super higher viremia titer (10^8^ pfu), as VP1-145G viruses were tested by Kobayashi et al.^41^

When assessing differences arising from the routes of viral infection (I.C. vs. I.V.), the CDV-Isehara models displayed no significant difference in disease incidence, survival rate or body weight change (Fig 5d2–5d4), although differences in the distribution of the virus in the tissues of infected KI mice were observed (Figs. 6b, 6d). The replication of CDV-Isehara was detected in all tested tissues of mice via the I.V. route, which differed a report by Fujii et al. in which the Isehara/Japan/99 strain was only detected in CNS in another Tg mouse model when using the same I.V. infection route. However, the dose used by Fujii et al. was 1 × 10^6^ TCID_50_^19^, whereas the dose used in our study was about 100 times higher. Therefore, the results obtained by Fujii et al. cannot be directly compared with those obtained in this study. It should be kept in mind that pathological tropisms observed in the CNS and peripheral sites cannot only be distinguished by clinical symptoms. The identification of virus distribution and viral load in different tissues still needs a combination of different assays used in this study.

In contrast to the CDV-Isehara models, significant differences between the CMU4232/I.C. and CMU4232/I.V. group were observed with respect to disease incidence, body weight change (Fig 5c2, c4) and the distribution of the virus in tissues (Figs. 6a, c). CMU4232 could only replicate in the brains of mice when 3.2–6.7 × 10^6^ pfu was administered via the I.C. route. However, via an I.V. administration of 3.2–7.5 × 10^8^ pfu, CMU4232 could infect multiple organs. Following viremia, CMU4232 disseminated from the peripheral to central, leading to the observed CNS manifestations finally and demonstrating that the viral dose/hyperviremia is crucial to the in pathogenicity of EV-A71. Our results are in agreement with a recent study in which epidemic Chinese EV-A71 strains isolated from 2008–2010 were compared in neonatal ICR mice and neonatal rhesus monkeys^27^. It appears that the CMU4232/IV mouse model mimics not only the neurological manifestation and infection of multiple organs injury following hyperviremia but also the morbidity of EV-A71 infection in human beings to some extent. A previous study reported that the risk of neurological complications was 1.1% and the severe-case fatality risk was 3.0%, with >90% of deaths associated with EV-A71 in the research of HFMD in China from 2008–2012^9^.

In this study, we also compared the replication capacity of the strains CMU4232 and CDV-Isehara in RD cells through a plaque-forming assay. The growth curves showed that CDV-Isehara replicated faster and generated more virions than CMU4232 (Fig. 1c). As reported by Sun et al., a difference in the replication capacity of EV-A71 strains in RD cells could indicate their pathogenicity in humans^43^.

There appeared to be some differences in viral loads of the two strains detected in vitro and in vivo. Under the I.C. inoculation, the virus CDV-Isehara was detected in different tissues including the brain, intestine, muscle, and heart (Fig. 6b) whereas the virus CMU4232 was only detected in the brain (Fig. 6a). Furthermore, the viral load in tissues infected by CDV-Isehara was higher than that infected by CMU4232. The finding was consistent to the growth curves in RD cells (Fig. 1c). Under the I.V. inoculation, both viruses were detected in the brain, heart, lung, intestine, and muscle (Figs. 6c,d). The highest viral loads of both viruses were observed in muscles, dissimilar to those observed under the I.C. route infection. In addition, the viral load in tissues infected by CMU4232 was higher than that by CDV-Isehara. However, we did not observe differences in rates of morbidity and mortality and in change of the body weight of mice between these two viruses after I.V. inoculation. In contrast, CDV-Isehara-infected mice appeared to have a high proportion of severe symptoms compared with those of CMU4232-infected mice. The result indicated that the virus CDV-Isehara seems to be more virulent than the CMU4232 in the hSCARB2 KI mouse model. On the other hand, although having had high viral load in different tissues, the virus CMU4232 seemed to cause mild symptoms, suggesting that the virus CMU4232 is less virulent than that of CDV-Isehara. In short, one possible explanation for the difference observed may be related to the infectious routes used. Another explanation may be due to characteristics of the same strain in vitro and in vivo. In addition, we could not exclude the effect of timing of disease onset when tissue samples were taken from the infected mice.

EV-A71 strains have been shown to be continuously evolving. The strain CMU4232 used in this study is a member of the recent C4a-2 cluster, suggesting that CMU4232 could be a representative endemic strain after the HFMD outbreak in mainland China in 2008 (Fig. 3).

EV-A71 strains with the genotype C4 have been endemic and epidemic in mainland China for 20 years. The prevalence of neutralizing antibodies against the EV-A71 strains has been high in China and the selection pressure for the virus from population immunity is strong^44,45^. Indeed, as reported by Zhang et al. the evolution rate of C4a EV-A71 appeared to be faster than the average for all other EV-A71 genotypes^46^. Therefore, persistent attention should be paid to the changes in virulence resulting from evolution of EV-A71 viruses and their related pathogenic characteristics.

In summary, we showed that hSCARB2 KI mice are a sensitive and useful model for investigating the clinical outcomes caused by different EV-A71 strains. Diverse clinical symptoms characterized as bi-pathological tropism in the CNS and peripheral sites can be observed even when the “representative” Chinese strain CMU4232 was tested. The intracranial infection model appears to be suitable for evaluating of neurovirulence caused by EV-A71, whereas the venous infection model is useful for studying the pathogenicity of EV-A71. Moreover, impact of the change in key amino acids site (e.g., VP1-145) on pathogenesis in different hosts should be taken into account in future studies.

## Materials and methods

### hSCARB2 KI mice and ethics statements

The human SCARB2 KI mice^24^ used in this study were provided by the Institute for Laboratory Animal Resources, National Institute for Food and Drug Control, Beijing, China. The animal experiments were performed according to the recommendations in the national guidelines for the care and use of animals in scientific research “Regulations for the Administration of Affairs Concerning Experimental Animals”. The study protocol was approved by the Capital Medical University Animal Experiments and Experimental Animals Management Committee (AEEI-2016-121). Termination of mice was performed under anesthesia, and all efforts were made to minimize suffering. All samples used in this study were anonymized.

### EV-A71 viruses and cells

CMU4232 (EV71/CMU4232-1/BJ/CHN/2008) was originally isolated from a HFMD patient by our group during the 2008 outbreak in Beijing^8^, and it was passaged in RD cells (National Infrastructure of Cell Line Resource, Beijing, China) and stocked in our laboratory. An infectious cDNA clone of EV-A71 (pSVA14-Isehara ver4) was kindly provided by Professor Satoshi Koike, Tokyo Metropolitan Institute of Medical Science, Tokyo, Japan. The RD cell line was cultured in DMEM containing 4.5 g/L glucose, L-glutamine, and sodium pyruvate (Corning, 10-013-CVR, USA) supplemented with 10% fetal bovine serum (FBS) (Corning, 35-076-CV, USA), 100 IU of penicillin, and 100 μg of streptomycin per ml. The cells were incubated at 37 ℃ and with 5% CO_2_. CMU4232 and the rescued virus, CDV-Isehara were harvested from RD cultures by freezing and thawing three times and were stored at −80 ℃. The titers of the virus stocks were tested using a modified plaque-forming assay and determining the CCID50.

### Growth curves of CMU4232 and CDV-Isehara in RD cells

To assess the proliferation dynamics of both EV-A71 strains, the growth curves were determined as follows: RD cells cultured in 24-well plates were inoculated at an MOI of 1 or 0.1 in triplicate. After being allowed to attach for 2 h, the unbound viral particles were washed off with PBS. Next, the infected cells were cultured at 37 ℃ for 0, 8, 24, 48, 72, and 96 h and were titrated to determine pfu values after three consecutive freeze–thaw cycles. All assays were performed in triplicate.

### Western blot analysis

Infected RD cells were collected and lysed using RIPA buffer. Viral and cellular proteins were separated by SDS-PAGE on a 12% gel and were electrotransferred onto a PVDF membrane. After blocking, the membrane was probed with a mouse anti-EV-A71 antibody (Millipore, MAB979, 1:1000 dilution) and an HRP-conjugated secondary antibody (Transgen Biotech, HS201-1, 1:5000 dilution).

### Reverse transcription (RT)-PCR and nucleotide sequencing of CMU4232

Viral RNA of the CMU4232 strain was extracted from infectious RD cell supernatant using a QIAmp^®^ Viral RNA Kit (Qiagen, Hilden, Germany). RNA was reverse transcribed into cDNA using a Superscript II Kit (Invitrogen, USA) according to the manufacturer’s instructions. All sequencing was performed by Guangzhou Darui Biotechnology Co., Ltd. The accession number of the complete genome sequence of CMU4232 is MH373639.

### Nucleotide and deduced amino acid sequence identity analysis between CMU4232 and CDV-Isehara

The complete genome sequence alignment of CMU4232 and CDV-Isehara was performed using the Bioedit and Clustal W programs in MEGA 4.0, and the deduced amino acid sequences of structural and nonstructural proteins were aligned and shown.

### Phylogenetic analysis

Representative strains of each EV-A71 subgenotypes were chosen based on a previously published study, especially representative strains available at the Genbank reported in mainland China that were first screened from those isolated from different regions and time periods from 1998 to 2017. Phylogenetic analysis was based on viral VP1 protein-coding nucleotide sequences, and the phylogenetic tree was constructed using the neighbor-joining method using MEGA 4.0 software with 1000 bootstrap replications. Details of all the EV-A71 strains included in the phylogenetic tree are provided in Supplementary Table S2.

### Infection of EV-A71 in hSCARB2 KI mice

To evaluate the susceptibility of the hSCARB2 KI mice to EV-A71 infection, we challenged 1-, 2-, 3-, 4-, and 6-week-old KI mice I.C. with the CDV-Isehara strain at a dose of 4.8 × 10^6^ pfu and observed the development of clinical symptoms daily for 2 weeks.

In addition, we chose 3-week-old hSCARB2 KI mice to be inoculated with EV-A71 (CMU4232/ CDV-Isehara) at the indicated doses and routes (I.C. infection at a dose of 3.2–6.7 × 10^6^ pfu or I.V. infection at a dose of 3.2–7.5 × 10^8^ pfu per animal). Control mice were mock injected with an equivalent volume of RD cell lysate supernatant. All mice were monitored daily for clinical signs, survival, and body weight for 2 weeks. The activity level, mental status, degree of limb paralysis, and other symptoms were observed and recorded daily for each mouse. Furthermore, the clinical signs were scored as follows: asymptomatic (0), ruffled fur and/or hunchbacked appearance (1–2), easily frightened/ trembling/shrinking/mental bluntness/dispirited/slow in action (3–5), limb-shake weakness (6), wasting > 3 consecutive days (7), limb paralysis (8), and moribund and death (9). Based on the scores, we classified the clinical manifestations at four levels, asymptomatic (0–2); mild symptoms (3–5); severe symptoms (6–8); and death (9). Experiments for group of 3-week-old mice were performed in triplicate.

### Quantitative detection of EV-A71 RNA by real-time RT-PCR in different tissues

The infected mice were killed at the peak onset of disease development and were immediately processed for sample collection. Tissues were collected, including brains, hearts, lungs, skeletal muscle, and intestines. Total RNA was extracted from different tissues using TRIzol reagent (Invitrogen, 15596026, USA) following the manufacturer’s instructions, and then was converted into cDNA with a RT-PCR kit (Transgen Biotech, AE301-02) followed by qPCR using a PCR system (Transgen Biotech, AQ101-02). PCR amplifications were performed at 94 ◦C for 30 s followed by 40 cycles of 94 ◦C for 5 s, 60 ◦C for 15 s, and 72 ◦C for 10 s. SYBR Green PCR Master Mix (Transgen Biotech, AQ131-02) was used to detect of viral load in different tissues using the primers of EV71-S /A (S3 Table). Mouse β-actin cDNA was used as an internal control. Each assay was carried out in triplicate.

### Histopathology examination (HE) and immunohistochemistry (IHC) analysis

Tissue samples from control and EV-A71-infected hSCARB2 KI mice were fixed with formalin buffered saline by perfusion and then dehydrated and embedded in paraffin and sectioned according to conventional procedures. Some sections were either stained with hematoxylin–eosin or were used for IHC analysis. Tissue sections were dewaxed and rehydrated in graded ethanol, and IHC was performed using a standard avidin–biotin immunoperoxidase technique. An anti-EV-A71 monoclonal antibody (MAB979; Millipore, USA), was used as a primary antibody.

## Electronic supplementary material

Supplementary Tables
