# Supplementary material for: Severity of enterovirus A71 infection in a human SCARB2 knock-in mouse model is dependent on infectious strain and route
Source: Emerg Microbes Infect. 2018 Dec 5;7:205. doi: 10.1038/s41426-018-0201-3 (PMC6281673; doi:10.1038/s41426-018-0201-3)
Supplement: Supplementary file 1 — Supplementary Tables [file 41426_2018_201_MOESM1_ESM.docx]

**Supporting information**

**S1 Table. Pairwise nucleotide and amino acid sequences identities between CMU4232 and CDV-Isehara**

| **Region/%Identities** | **VP1** | **VP2** | **VP3** | **VP4** | **2A** | **2B** | **2C** | **3A** | **3B** | **3C** | **3D** |
| --- | --- | --- | --- | --- | --- | --- | --- | --- | --- | --- | --- |
| **nucleotide** | 89 | 90.8 | 89.4 | 89.4 | 83.1 | 74.1 | 80.7 | 76.4 | 74.2 | 76.7 | 76.6 |
| **amino acid** | 98.3 | 99.6 | 100 | 100 | 95.3 | 92.9 | 97 | 95.3 | 90.9 | 91.8 | 93.5 |

**S2 Table. The list of EV-A71 strains used for phylogenetic analysis in this study**

| **Strain** | **Year of isolation** | **Place of isolation** | **Geno-group** | **GenBank no.** |
| --- | --- | --- | --- | --- |
| **SHZH98-CHN-1998** | 1998 | GD /CHN | C4 | AF302996 |
| **AFP2001064-GX-CHN-2001** | 2001 | GX/CHN | C4 | JQ742001.1 |
| **SHH02-17-SH-CHN-2002** | 2002 | SH/CHN | C4 | AY547500 |
| **CQ03-1-CHN-2003** | 2003 | CQ/ CHN | C4 | AY547501 |
| **SHZH04-3-CHN-2004** | 2004 | GD /CHN | C4 | AY895142 |
| **05488-SD-CHN-2005** | 2005 | SD/ CHN | C4 | GQ253421 |
| **06282-SD-CHN-2006** | 2006 | SD/ CHN | C4 | GQ253423 |
| **4211-BJ-CHN-2007** | 2007 | BJ/CHN | C4 | EU024958 |
| **CMU0804-BJ-CHN-2008** | 2008 | BJ/CHN | C4 | JX297495 |
| **CMU4232** | 2008 | BJ/CHN | C4 | MH373639 |
| **Nanjing-JS06-CHN-2009** | 2009 | JS/CHN | C4 | GU353080 |
| **1111-GD-CHN-2010** | 2010 | GD/ CHN | C4 | JF519718 |
| **cx005-Ningbo-ZJ-CHN-2011** | 2011 | ZJ/ CHN | C4 | JQ284022 |
| **SJZ2012-0985T-HeB-CHN-2012** | 2012 | HeB/CHN | C4 | KY081981.1 |
| **A006-GX-2013** | 2013 | GX/CHN | C4 | MF185258.1 |
| **BJ-2-2014** | 2014 | BJ/CHN | C4 | KU710722.1 |
| **ZJ01-CHN-2015** | 2015 | ZJ/ CHN | C4 | KY406804.1 |
| **SH2016-067-CHN-2016** | 2016 | SH/CHN | C4 | KX8716770 |
| **CZTN01-CHN-2017** | 2017 | JS/CHN | C4 | MG431943 |
| **BrCr-USA-1970** | 1970 | USA | A | U22521 |
| **242-TW-1986** | 1986 | TW | B1 | JN874548 |
| **Y90-3205-JPN-1990** | 1990 | JPN | B2 | AB433863 |
| **26M-AUS-1999** | 1999 | AUS | B3 | AF376101 |
| **E59-TW-2002** | 2002 | TW | B4 | JN874551 |
| **EV1945-Kuching-MAL-2009** | 2009 | MAL | B5 | HM358835 |
| **P38-Sm-W-JPN-2000** | 2000 | JPN | C1 | HQ676217 |
| **JP53-Yg-W-JPN-1998** | 1998 | JPN | C2 | HQ676232 |
| **CDV-Isehara( Isehara/Japan/99)** | 1999 | JPN | C2 | LC375764.1 |
| **01-KOR-2000** | 2000 | KOR | C3 | AY125966 |
| **07364-TW-2007** | 2007 | TW | C5 | EU527983 |

There are 30 complete VP1 sequences of EV-A71 strains were involved in this study totally. The EV-A71 strains from different isolation regions of mainland China, were selected according to available time distribution.

Abbreviation of Chinese cities: BJ, Beijing；SH, Shanghai; CQ, Chongqing. Abbreviation of Chinese provinces: SD, Shandong; GD, Guangdong; GX, Guang Xi; ZJ, Zhejiang; JS, Jiangsu; HeB, Hebei;

Region abbreviation: TW, Taiwan.

Country abbreviations: AUS, Australia; KOR, Korea; JPN, Japan; MAL, Malaysia; USA, United States of America; CHN, People’s Republic of China.

**S3 Table. Primers of EV71-S /A used in qPCR for detection of viral load in different tissues**

| **QP71-F** | 5-AGTGATGAGAGTATGATTGAGACACG-3 |
| --- | --- |
| **QP71-R** | 5-CCCGCTCTGCTGAAGAAACT-3 |
